# Training thermodynamic computers by gradient descent

**DOI:** 10.1073/pnas.2528413123

**Published:** 2026-04-03

**Authors:** Stephen Whitelam

**Affiliations:** ^a^Molecular Foundry, Lawrence Berkeley National Laboratory, Berkeley, CA 94720

**Keywords:** statistical mechanics, thermodynamic computing, machine learning

## Abstract

We show how to adjust the parameters of a thermodynamic computer by gradient descent in order to perform a desired computation at a specified observation time. Within a digital simulation of a thermodynamic computer, training proceeds by maximizing the probability with which the computer would generate an idealized dynamical trajectory. The idealized trajectory is designed to reproduce the activations of a neural network trained to perform the desired computation. This teacher-student scheme results in a thermodynamic computer whose finite-time dynamics enacts a computation analogous to that of the neural network. The parameters identified in this way can be implemented in the hardware realization of the thermodynamic computer, which will perform the desired computation automatically, driven by thermal noise. We demonstrate the method on a standard image-classification task, and estimate the thermodynamic advantage—the ratio of energy costs of the digital and thermodynamic implementations—to exceed seven orders of magnitude. Our results establish gradient descent as a viable training method for thermodynamic computing, enabling application of the core methodology of machine learning to this emerging field.

## Significance

As modern computing becomes limited by energy consumption, there is growing interest in physical computing paradigms that can operate closer to fundamental thermodynamic limits. Thermodynamic computing is an emerging field in which computation is done by the natural time evolution of a physical system in contact with a thermal bath, rather than by explicit simulation on a digital computer. Nonlinear thermodynamic computers can in principle perform nonlinear computations similar to those performed by classical neural networks, but with much less energy consumption. The contribution of the present paper is to show how a thermodynamic computer can be trained to perform such a task using gradient descent, thereby opening the door to the training of thermodynamic computers for large-scale problems.

Thermodynamic computing offers a potential route to energy-efficient computation. Unlike digital or quantum computing, which must at considerable energetic cost overpower or suppress thermal noise, thermodynamic computing is designed to use thermal noise as a source of energy. Physical devices whose states evolve under Langevin dynamics can be engineered to perform computations as they relax toward thermal equilibrium. Because these computations are carried out by the natural dynamics of the system, such devices can in principle operate with very low energy overhead, approaching fundamental thermodynamic limits (1–6).

A key challenge for thermodynamic computing is to identify algorithms that make efficient use of thermodynamic hardware and that reproduce the algebraic and machine-learning operations done digitally. Recent work has shown that thermodynamic computers can solve linear algebra problems, such as matrix inversion, in thermodynamic equilibrium (4, 5). The advantage of equilibrium operation is that the computer’s degrees of freedom obey the Boltzmann distribution, which depends in a precise way on the computer’s potential energy. By choosing this potential energy appropriately, therefore, we can specify the desired computation. In this mode of operation the device functions as a Boltzmann computer, sampling the Boltzmann distribution.^*^ However, one drawback of equilibrium operation is the requirement to attain equilibrium: Physical systems can take a long time to equilibrate (9, 10), and the equilibration time of thermodynamic computer programs can vary by orders of magnitude as the computer’s parameters are changed (11).

One response to slow equilibration is to devise ways of increasing the basic clock speed of a thermodynamic computer, such as by rescaling its potential energy and injecting noise in suitable proportion (11). Another is to design a thermodynamic computer to operate out of equilibrium. In recent work, we showed that a thermodynamic computer can be trained to perform a desired computation at a specified observation time, regardless of whether equilibrium has been attained (12, 13). In this mode of operation the device functions as a Langevin computer, performing computations via the properties of finite-time Langevin trajectories.

In ref. 12, we used a genetic algorithm to identify the parameters of a nonlinear thermodynamic computer capable of performing standard machine-learning computations at finite observation times. While effective, genetic algorithms are slower than gradient descent, the standard training method of machine learning (14, 15). The contribution of the present paper is to describe how to train a thermodynamic computer by gradient descent, in order to do calculations on a clock. In doing so, we enable application of the core training method of machine learning to thermodynamic computing, and confirm that thermodynamic computers can be operated on a clock, as conventional computers are. Training is done on a digital computer; the resulting parameters can be implemented in a hardware realization of the thermodynamic computer, which will perform the desired computation automatically, driven by thermal noise.

Our training method uses a teacher-student setup within a digital simulation of a thermodynamic computer. By taking derivatives of the Onsager-Machlup functional, the path weight for Langevin trajectories, we maximize the likelihood that the thermodynamic computer generates trajectories that reproduce the activations of a reference model, a trained neural network. In this way, we design the dynamics of the thermodynamic computer to perform calculations analogous to those performed by the neural network. We demonstrate the method by training a thermodynamic computer to perform image recognition at a specified observation time.

The authors of ref. 16 recently introduced a teacher-student scheme for thermodynamic computing based on quantum thermal machines coupled to reservoirs at different temperatures. Their quantum units implement Boolean logic gates by relaxing to nonequilibrium steady states. By contrast, our framework uses a network of classical, nonlinear units in contact with a single thermal bath. The parameters of this network are trained by gradient descent so that its finite-time, clocked dynamics reproduces the activations of a nonlinear neural network. This approach enables classical, programmable thermodynamic computing capable of tackling high-dimensional tasks such as image recognition, extending the scope of thermodynamic computing beyond Boolean logic (and linear algebra) to scalable machine learning.

## 1. Training a Thermodynamic Computer by Gradient Descent

Our model of a thermodynamic computer consists of *N* classical, real-valued fluctuating degrees of freedom $x=\{x_{i}\}$. These degrees of freedom can represent different physical observables in thermodynamic computers realized in hardware, including voltage states in electrical circuits (5), oscillator positions in a mechanical device (17), or phases in a Josephson junction (18, 19). The computer’s units $x_{i}$ evolve in time according to the overdamped Langevin dynamics

[1] $$\begin{array}{c} {\dot{x}}_{i}=-\mu \partial_{i}V_{\theta }\left(x\right)+\sqrt{2\mu k_{\text{B}}T}\;\eta_{i}\left(t\right). \end{array}$$

Here *μ* is the mobility parameter, which sets the basic time constant of the computer. For the thermodynamic computers of refs. 4 and 5, $\mu^{-1}$ is of order a microsecond. For damped oscillators made from mechanical elements (17) or Josephson junctions (18, 19), $\mu^{-1}$ is of order a millisecond or a nanosecond, respectively.

The first term on the right-hand side of Eq. **1** is the force arising from the computer’s potential energy $V_{\theta }\left(x\right)$, given a set of parameters (couplings and biases) $\theta =(\left\{J_{\mathrm{ij}}\right\},\left\{b_{i}\right\})$; note that $\partial_{i}\equiv \partial /\partial x_{i}$. These are the parameters we wish to adjust in order to have the computer perform a desired calculation. The second term on the right-hand side of Eq. **1** models thermal fluctuations: $k_{\text{B}}T$ is the thermal energy scale, and the uncorrelated Gaussian white noise terms satisfy $\langle \eta_{i}\left(t\right)\rangle =0$ and $\left\langle \eta_{i}\left(t\right)\eta_{j}\left(t^{\prime }\right)\right\rangle =\delta_{\mathrm{ij}}\delta \left(t-t^{\prime }\right)$.

We take the potential energy $V_{\theta }\left(x\right)$ of the computer to be

[2] $$\begin{array}{c} V_{\theta }\left(x\right)=\sum_{i=1}^{N}\left(J_{2}x_{i}^{2}+J_{4}x_{i}^{4}\right)+\sum_{i=1}^{N}b_{i}x_{i}+\sum_{\left(ij\right)}J_{\mathrm{ij}}x_{i}x_{j}. \end{array}$$

The first sum in Eq. **2**, which runs over the *N* units of the computer, sets the response of its individual degrees of freedom. For $J_{2}>0,J_{4}=0$ we have a linear model similar to that of ref. 4, whose activations in equilibrium are linear functions of their inputs. For $J_{2},J_{4}>0$^†^ we have a nonlinear model that is the thermodynamic analog of a neural network (12). This is the case we focus on in this paper. In general, it is not necessary for the computer’s potential to be exactly quartic: We can have a nonlinear model with any nonquadratic potential that is thermodynamically stable (bounded from below). We can consider Eq. **2** to represent a low-order Maclaurin expansion, in powers of $x_{i}$, of the potential of such a device.

The remaining terms in Eq. **2** contain the adjustable parameters of the computer. The parameters $b_{i}$ are input signals or biases applied to each unit. The parameters $J_{\mathrm{ij}}$ are pairwise couplings between units, and are motivated by the bilinear interactions of the thermodynamic computers of refs. 4 and 5. As a consequence, the information flow between units *i* and *j* is bidirectional—units *i* and *j* feel the forces $-J_{\mathrm{ij}}x_{j}$ and $-J_{\mathrm{ij}}x_{i}$, respectively—unlike in a feedforward neural network, whose connections are unidirectional.

Our goal is to use digital simulation to train a nonlinear thermodynamic computer, the thermodynamic analog of a neural network, to mimic the behavior of a neural network trained to perform a desired computation. The parameters identified in this way can then be implemented in the hardware realization of the thermodynamic computer, which can perform the desired computation automatically, driven by thermal noise.

**Fig. 1.**
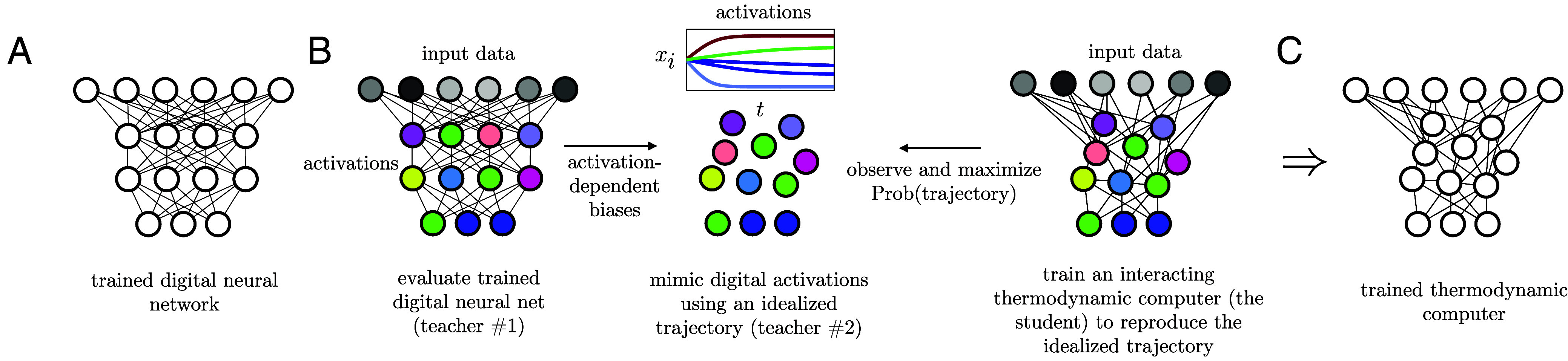
Training a thermodynamic computer by gradient descent. (*A*) A trained neural network is used as a reference model. (*B*) *Left*: Input data are passed through the neural network, producing node activations $A_{i}$ (colored circles, “teacher #1”). Center: These activations are the targets for an idealized trajectory $\left\{x_{i}^{\left(0\right)}\left(t\right)\right\}$ (“teacher #2”), such that the values $x_{i}^{\left(0\right)}\left(t_{\text{f}}\right)$ observed at time $t_{\text{f}}$ resemble the $A_{i}$. *Right*: An interacting thermodynamic computer (the “student”) is trained to maximize the probability of generating this trajectory, using the gradient-descent Eqs. **7** and **8**. (*C*) Repeating this process for many input data yields a trained thermodynamic computer, which mimics the behavior of the neural network.

Our proposed training scheme is shown in Fig. 1. As sketched in panel (*A*), we start with a neural network trained for a desired task. The neural network has *N* nodes, the same number of degrees of freedom as our thermodynamic computer, but with potentially different connectivity. We take the trained neural network (“teacher #1”), evaluate it for a particular set of input data, and record the *N* activations $A_{i}$ of its degrees of freedom. This step is shown on the *Left* of panel (*B*).

Next, we construct by hand an idealized dynamical trajectory $\left\{x_{i}\left(t\right)\right\}$ of the thermodynamic computer. This step is shown in the *Center* of panel (*B*). In this idealized trajectory the *N* activations $x_{i}$ of the thermodynamic computer evolve with time toward values equal to (or similar to) the activations $A_{i}$ of the trained digital computer. There are different ways to construct such a trajectory. A natural way to do so is to use a noninteracting thermodynamic computer, setting $J_{\mathrm{ij}}^{\left(0\right)}=0$ and taking the hidden and output biases $b_{i}^{\left(0\right)}$ proportional to the corresponding activations $A_{i}$ of the trained neural network. The idealized trajectory can even be deterministic (e.g. we can set the temperature of the noninteracting reference computer to zero, $T^{\left(0\right)}=0$): As long as we calculate the probability with which a noisy, finite-temperature thermodynamic computer generates the idealized trajectory, the latter can be constructed for convenience.

Ultimately, our goal is to have the trained thermodynamic computer reproduce the neural-network outputs, but we also require the computer to learn the net’s hidden activations. The neural network relies on its hidden units to compute its outputs, and so we reason that reproducing its hidden-unit behavior will allow the thermodynamic computer to acquire a computational capacity similar to that of the neural network.

This idealized trajectory (“teacher #2”) is observed by an interacting thermodynamic computer (or “student”), the device we wish to train. The student computer, which operates at finite temperature *T* and has connections $J_{\mathrm{ij}}$ and biases $b_{i}$, sees the same input data that produced the activations $A_{i}$ of the trained neural network. As the teacher trajectory runs, the parameters of the student thermodynamic computer are adjusted by gradient descent in order to increase the likelihood with which it *would* generate the idealized trajectory. This step, explained in detail below, is shown on the *Right* of panel (*B*) of Fig. 1.

The training step shown in panel (*B*) is repeated for many input examples. The result is a trained thermodynamic computer, illustrated in panel (*C*), whose finite-time dynamics realizes a computation analogous to that carried out by the trained neural network. The computer’s trained parameters θ could then be realized in hardware, encoding the forces that drive the computer to perform the same computation as the neural network in panel (*A*). When presented with input data, the hardware thermodynamic computer will evolve automatically, driven by thermal noise.

### 1.1. Details of the Gradient-Descent Algorithm.

The gradient-descent algorithm sketched in Fig. 1*B* is designed to adjust the parameters $\theta =(\left\{J_{\mathrm{ij}}\right\},\left\{b_{i}\right\})$ of an interacting thermodynamic computer in order to maximize the likelihood with which it would generate an idealized trajectory, $\omega ={\left\{x\left(t_{k}\right)\right\}}_{k=0}^{K}$, observed at discrete times $t_{k}=k\Delta t$; note that $K=\left\lfloor t_{\text{f}}/\Delta t\right\rfloor$. The idealized trajectory is designed to make the activations $x_{i}\left(t_{\text{f}}\right)$ of the thermodynamic computer at some observation time $t_{\text{f}}$ equal to (or similar to) the activations $A_{i}$ of a trained neural network (*Left*). Starting with the parameters of the trained computer set to zero, θ=0, we proceed as follows.

On a digital computer we integrate Eq. **1** according to a discretized update equation, such as the first-order Euler scheme

[3] $$\begin{array}{c} x_{i}\left(t+\Delta t\right)=x_{i}\left(t\right)-\mu \partial_{i}V_{\theta }\left(x\right)\Delta t+\sqrt{2\mu \;k_{\text{B}}T\Delta t}\;\eta_{i}. \end{array}$$

Here Δt is the integration timestep, and $\eta_{i}∼N\left(0,1\right)$ is a Gaussian random variable with zero mean and unit variance. We define $\Delta x_{i}\left(t\right)\equiv x_{i}\left(t+\Delta t\right)-x_{i}\left(t\right)$. Under this integration scheme, the probability of generating a step x→x+Δx of the idealized trajectory is that of drawing the *N* noise values $\eta_{i}$, which is

[4] $$\begin{array}{c} P_{\theta }^{\mathrm{step}}\left(\Delta x\right)={\left(2\pi \right)}^{-N/2}\prod_{i=1}^{N}\exp\left(-\eta_{i}^{2}/2\right), \end{array}$$

where the $\eta_{i}$ are specified by Eq. **3**. We can solve Eq. **3** for $\eta_{i}$ to give

[5] $$\begin{array}{c} \eta_{i}=\frac{\Delta x_{i}+\mu \partial_{i}V_{\theta }\left(x\right)\Delta t}{\sqrt{2\mu k_{\text{B}}T\Delta t}}, \end{array}$$

and insert [5] into [4]. The resulting expression is the probability that a computer with parameters θ would have generated the step x→x+Δx. The negative logarithm of this expression is

[6] $$\begin{array}{c} -\ln P_{\theta }^{\mathrm{step}}\left(\Delta x\right)=\sum_{i=1}^{N}\frac{{\left(\Delta x_{i}+\mu \;\partial_{i}V_{\theta }\left(x\right)\Delta t\right)}^{2}}{4\mu k_{\text{B}}T\Delta t}, \end{array}$$

up to an unimportant constant term. Eq. **6** is a discrete version of the Onsager-Machlup action associated with the Langevin equation (21, 22).

The negative log-probability $-\ln P_{\theta }\left(\omega \right)$ with which the computer would generate the entire idealized trajectory $\omega ={\left\{x\left(t_{k}\right)\right\}}_{k=0}^{K}$ is then a sum of terms [**6**] over each step of the trajectory, $-\ln P_{\theta }\left(\omega \right)=-\sum_{k=0}^{K-1}\ln P_{\theta }^{\mathrm{step}}\left(\Delta x\left(t_{k}\right)\right)$. This is our loss function. To increase the likelihood with which the thermodynamic computer would generate that trajectory, we update each parameter of the computer by the gradient of the loss with respect to itself, giving

[7] $$\begin{array}{cc} J_{\mathrm{ij}} & \to J_{\mathrm{ij}}+\alpha \sum_{k=0}^{K-1}\frac{\partial }{\partial J_{\mathrm{ij}}}\ln P_{\theta }^{\mathrm{step}}\left(\Delta x\left(t_{k}\right)\right), \end{array}$$

[8] $$\begin{array}{cc} b_{i} & \to b_{i}+\alpha \sum_{k=0}^{K-1}\frac{\partial }{\partial b_{i}}\ln P_{\theta }^{\mathrm{step}}\left(\Delta x\left(t_{k}\right)\right), \end{array}$$

where *α* is a learning rate. The gradient terms in Eqs. **7** and **8** can be calculated analytically, using Eq. **2**, and are

[9] $$\begin{array}{cc} -\frac{\partial }{\partial J_{\mathrm{ij}}}\ln P_{\theta }^{\mathrm{step}}\left(\Delta x\right) & =\frac{\Delta x_{i}+\mu \;\partial_{i}V_{\theta }\left(x\right)\Delta t}{2k_{\text{B}}T}x_{j}\\ & +\frac{\Delta x_{j}+\mu \partial_{j}V_{\theta }\left(x\right)\Delta t}{2k_{\text{B}}T}x_{i}, \end{array}$$

and

[10] $$\begin{array}{c} -\frac{\partial }{\partial b_{i}}\ln P_{\theta }^{\mathrm{step}}\left(\Delta x\right)=\frac{\Delta x_{i}+\mu \partial_{i}V_{\theta }\left(x\right)\Delta t}{2k_{\text{B}}T}, \end{array}$$

where

[11] $$\begin{array}{c} \partial_{i}V_{\theta }\left(x\right)=2J_{2}x_{i}+4J_{4}x_{i}^{3}+b_{i}+\sum_{j\in N(i)}J_{\mathrm{ij}}x_{j}. \end{array}$$

Here N(i) denotes the set of units connected to unit *i*. All quantities $x_{i}$ in Eqs. **9**–**11** are evaluated at time $t_{k}$, appropriate for each term in Eqs. **7** and **8**.

The thermodynamic computer that we are training to reproduce the idealized trajectory sees the same input data as the neural network whose activations we are seeking to copy. This can be accomplished by viewing the input data as a set of frozen variables $x_{i}$, and coupling them to the degrees of freedom $x_{j}$ of the thermodynamic computer via the trainable interactions $J_{\mathrm{ij}}x_{i}x_{j}$ (in this respect the input data function as a set of biases). We repeat the process for a range of input data, and record the resulting parameters θ of the thermodynamic computer.

Note that the teacher thermodynamic computer is a computational construct that is not designed to be realized in hardware. For one, its dynamics is guided by the hidden and output activations of the neural network, and so we must operate the neural network in order to run the teacher computer. For another, we can run it in the absence of noise, which is not true of hardware thermodynamic computers. The trained thermodynamic computer *is* designed to be realized in hardware. It is run at finite temperature, just as the corresponding hardware would be. In addition, it does not require input from the neural network. The point of training is to identify the couplings $J_{\mathrm{ij}}$ that allow its dynamics to perform computations given different input data, just as a neural network’s weights allow it to process multiple inputs without reparameterization.

Training is done by online gradient descent: After each idealized teacher trajectory is shown to the thermodynamic computer, we compute the gradient of the log-likelihood of that trajectory with respect to the model parameters, and update those parameters in the direction that increases that likelihood. The per-trajectory gradient is an unbiased estimate of the gradient of the average loss over all trajectories (23). In expectation, therefore, every update step follows the true gradient of the mean negative log-likelihood, and so the convergence guarantees of training should be those possessed by standard neural-network training: For a sufficiently small learning rate, the parameter updates should reduce the mean loss and converge to a stationary point of the objective. For a nonconvex surface that stationary point is not guaranteed to be a global optimum, and we must rely on empirical tests to determine how good the solution is.

## 2. Training a Thermodynamic Computer to Classify Images

To illustrate the method, we applied it to MNIST digit classification (24). To create the teacher model, we trained a fully connected neural network with two hidden layers of 32 nodes each, hyperbolic tangent activations, and 10 output nodes (one per digit class). Using stochastic gradient descent (learning rate 0.2, weight decay $10^{-4}$, batch size 256) for 300 epochs, the network achieved a test-set accuracy of 97.3%.

The thermodynamic computer consists of 64 hidden nodes and 10 output nodes. It therefore has the same number of degrees of freedom as the neural net, but is connected differently. The computer is connected by trainable couplings to the 784 pixels of the MNIST digit, whose values are shifted and scaled in order to have zero mean and unit variance (they are also further normalized to ensure that each digit has the same L2 norm). The computer has all-to-all connections between input data and hidden nodes, between input data and output nodes, between hidden nodes and output nodes, and within the set of hidden nodes. In all, it has 63,143 adjustable parameters. The intrinsic energy scale of the computer is set by the couplings $J_{2}=J_{4}=k_{\text{B}}T$; all energies will be displayed in units of $k_{\text{B}}T$.

We constructed idealized teacher trajectories using a noninteracting version of the thermodynamic computer $\left(J_{\mathrm{ij}}^{\left(0\right)}=0\right)$ run at zero temperature $\left(T^{\left(0\right)}=0\right)$. For a given MNIST digit, the guide biases $b_{i}^{\left(0\right)}$ of the teacher’s hidden units were set proportional to the activations $A_{i}$ of the neural network’s hidden nodes, while the guide biases $b_{i}^{\left(0\right)}$ of the output units were set proportional to the shifted activations $2A_{i}-1$ of the neural network’s output nodes. In this way, we wish to encourage the output units of the student computer to become positive when they recognize a digit of the relevant class, and negative otherwise. The long-time values of the activations under the teacher trajectories are not directly proportional to the values of the biases, because units’ intrinsic responses are nonlinear; in addition, the effective activation function of the thermodynamic computer is different in detail to the hyperbolic tangent function of the neural network (12). However, the teacher computer’s biases and long-time activations are strongly correlated, and we reason this is sufficient for a classifier, which depends ultimately on the hierarchies of the output activations and not their precise values. Activations were initially zero, $x_{i}\left(0\right)=0$, and the trajectory time was set to be $t_{\text{f}}=1/5$, in units of $\mu^{-1}$, the fundamental clock time of the computer. This time is short enough that the trained computer is out of equilibrium when observed, but long enough for unit activations $x_{i}\left(t_{\text{f}}\right)$ to become nonlinear functions of their inputs (12), so providing the expressiveness of a universal approximator.

The student computer was trained using multiple passes through a class-balanced version of the MNIST training set, slightly fewer than $10^{6}$ trajectories in all. Testing is done by taking the trained computer, showing it an MNIST test-set digit, identifying which output unit is most positive, and comparing that prediction with the digit’s class. The computer achieves a top-1 (respectively top-2) test-set accuracy of 92.0% (respectively 96.7%) on the standard MNIST test set, when averaged over 10 independent trajectories. For single trajectories, these numbers are respectively 91.7% and 96.5%, showing the computer’s calculation to be nearly deterministic, despite the stochastic nature of its dynamics.

There is a performance gap between the thermodynamic computer and the digital neural network on which it is trained. On MNIST, the computer’s accuracy is 95% (top-1) and 97% (top-2) of the accuracy of the neural network. We performed the same calculations using the Fashion-MNIST dataset (25) (using the same neural-net teacher and hyperparameters as for MNIST). The neural network achieves an accuracy on Fashion-MNIST of 84.9% (top-1) and 95.6% (top-2); the computer’s accuracy is 91% and 96% of that of the neural network. For larger and more complex datasets it is likely that this digital-thermodynamic performance gap will grow, and improvements in thermodynamic computer architecture and training methods will be required to do machine learning approaching the current digital state of the art. Digital neural networks required years of engineering improvements in order to reach current levels of performance (14, 15). Nonetheless, our results demonstrate the viability of gradient-descent training for doing machine-learning tasks by thermodynamic computing.

**Fig. 2.**
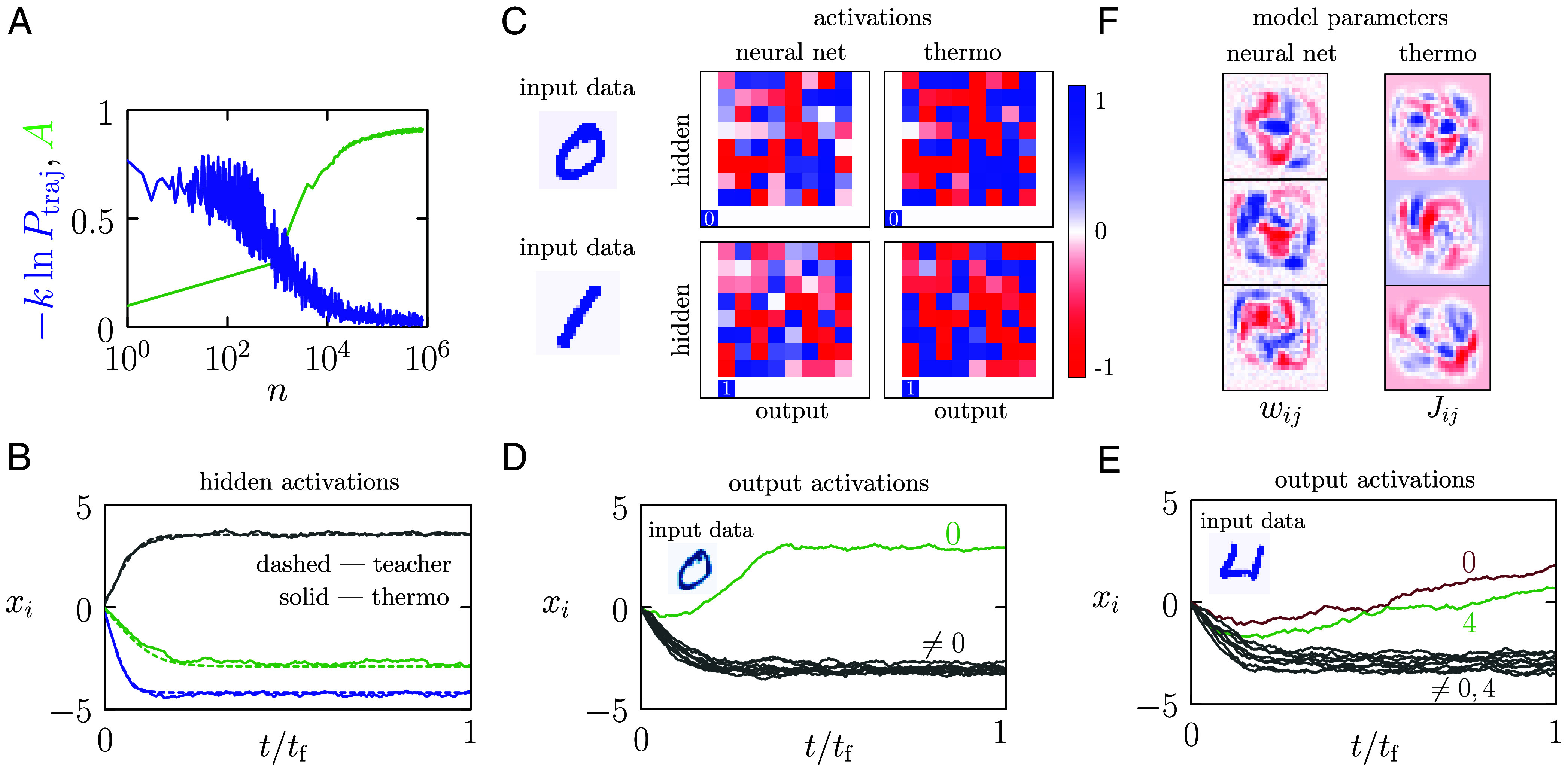
Training a thermodynamic computer to classify MNIST images. (*A*) The loss function, the negative log probability that the student thermodynamic computer generated the idealized training trajectory, as a function of the number *n* of training trajectories, for a 10-class image classifier ($k=10^{-3}$). We also show the validation-set accuracy *A* of the thermodynamic computer trained using *n* teacher trajectories. (*B*) Three activations of the trained computer as a function of time (solid lines, activations distinguished by the three colors), compared with their time dependence in the idealized teacher trajectory (dashed lines). (*C*) Hidden and output activations given two input digits, for the neural network (*Left*) and the trained thermodynamic computer observed at time $t_{\text{f}}$ (*Right*). The latter values are averaged over 5 trajectories. (*D* and *E*) Activations as a function of time for the thermodynamic computer when connected to the displayed digit. The example in (*D*) is correctly classified, while the example in (*E*) is not. (*F*) Trained neural network weights $w_{\mathrm{ij}}$ and thermodynamic computer couplings $J_{\mathrm{ij}}$ connecting the input data to 3 of the 64 hidden nodes.

Fig. 2*A* shows the loss function, the negative log probability with which the student computer would generate the idealized teacher trajectory, as a function of the number of training trajectories *n*. We generate one training trajectory for each digit observed, and pass repeatedly through the training set, updating the student computer’s parameters according to Eqs. **7** and **8** after each digit (called online learning). We also show the accuracy *A* of the partially trained student computer on a 1,000-digit subset of the test set, as a function of *n*. This information is not used during training.

Once the student computer is trained, we can observe its behavior when shown various MNIST digits. In Fig. 2*B* we show (solid lines) three of the trained thermodynamic computer’s activations $x_{i}$ as a function of time, when shown a particular training-set digit. The teacher trajectories are shown as dotted lines. The coincidence of the dotted and solid lines shows the computer to have learned to reproduce the teacher trajectories with reasonable accuracy. Panel (*C*) compares all the activations of the trained computer, at time $t_{\text{f}}$, with those of the neural network, for two training-set digits. The computer’s outputs are averaged over 5 independent trajectories. Blue and red pixels indicate positive and negative values, respectively. For ease of display on the color map, hidden activations are scaled so that they lie within the interval [−1,1], while output activations are shifted and scaled to lie within [0,1]. While not identical, the correlation between the thermodynamic computer’s activations and those of the trained neural network is clear.

In Fig. 2*D* we show how inference works during the operation of the trained thermodynamic computer. When shown the indicated digit, the three outputs of the computer adopt values showing that it predicts the digit, correctly, to be a 0. Panel (*E*) shows an example in which the computer misclassifies the digit (a 4) as a 0. Its output shows its decision to be split between those two classes. The computer is visibly out of equilibrium, its one-time quantities still evolving. The training process is designed to produce an answer at a specified time, whether or not the computer has attained equilibrium at that time.

Finally, Fig. 2*F* compares the neural-network weights $w_{\mathrm{ij}}$ with the thermodynamic computer couplings $J_{\mathrm{ij}}$ connecting the 784 input pixels to three representative hidden units. Despite being produced by different methods of training, the resulting filters are structurally similar: Both models discover localized, digit-specific contrast patterns that emphasize class-relevant strokes, while suppressing irrelevant background. This qualitative correspondence indicates that the thermodynamic computer learns internal feature detectors analogous to those of the neural network. One notable difference exists between the two plots: The couplings of the thermodynamic computer break symmetry and take both positive and negative values, reflecting the fact that its input pixels are centered symmetrically about zero.

The observation time $t_{\text{f}}$ can be chosen for convenience, provided that it is longer than the small basic relaxation time of the individual units of the computer (12). In particular, no part of the training scheme requires the computer to attain equilibrium, and so $t_{\text{f}}$ can be much shorter than the computer’s equilibration time. It may be, for a given choice of $t_{\text{f}}$, that the computer has time to equilibrate, but this is not necessary. In this example, we choose to train the computer to work at an observation time $t_{\text{f}}=0.2$ (in units of $\mu^{-1}$). Table 1 shows that the performance of the trained computer is largely insensitive to small variations of this choice, only dropping appreciably for very short observation times.

**Table 1.** Top-1 ($A_{1}$) and top-2 ($A_{2}$) test-set classification accuracies of the trained thermodynamic computer on MNIST as a function of observation time $t_{f}$, in units of the reciprocal Langevin mobility, $\mu^{-1}$

$t_{f}$	$A_{1}$	$A_{2}$
0.001	0.444	0.570
0.01	0.648	0.780
0.025	0.838	0.927
0.05	0.898	0.962
0.10	0.917	0.967
0.20	0.920	0.965
0.50	0.919	0.965

The computer is trained to do classification at an observation time $t_{\text{f}}=0.2$.

The computer’s energy scale relative to the thermal energy, $J_{2}/k_{\text{B}}T$, determines the basic scale of fluctuations of the degrees of freedom $x_{i}$. For classical computing, this ratio is many orders of magnitude, and so fluctuations of readout variables are strongly suppressed. Here, we set $J_{2}/k_{\text{B}}T=1$, to explore the regime in which the computer’s degrees of freedom fluctuate strongly; we have shown that computation is possible even under these conditions. In Table 2, we show the relative classification accuracies of the computer if run at different temperatures (or equivalently in the presence of nonthermal noise of the indicated scale). The operation of the computer remains robust up to noise levels multiple times that of the thermal energy.

**Table 2.** Top-1 ($A_{1}$) and top-2 ($A_{2}$) test-set classification accuracies of the trained thermodynamic computer on MNIST, for various temperatures

$k_{\text{B}}T/J_{2}$	$A_{1}/A_{1}^{\left(T=1\right)}$	$A_{2}/A_{2}^{\left(T=1\right)}$
0	1.0006	1.0001
0.01	1.0004	1.0001
0.1	1.0000	1.0005
1	1.0000	1.0000
2	0.9986	0.9999
5	0.9984	0.9981
10	0.9955	0.9965
25	0.9834	0.9886
100	0.9090	0.9457

Accuracies are shown relative to those at the training temperature of $k_{\text{B}}T/J_{2}=1$.

## 3. Energetic Considerations

Being able to train a thermodynamic computer to mimic a neural network enables low-energy machine learning in thermodynamic hardware. To quantify the *thermodynamic advantage*

[12] $$\begin{array}{c} A_{\mathrm{th}}\equiv \frac{E_{\mathrm{digital}}}{E_{\mathrm{thermo}}}, \end{array}$$

the ratio of energy costs of doing the computation digitally and thermodynamically, we can compare the energy scales of MNIST inference on digital and thermodynamic hardware. The following estimate refers only to the cost of calculation, and not of subsequently moving or processing the data.

The basic energy scale of a neural network is the cost of a multiply-accumulate (MAC) operation, in which two numbers are multiplied and added to a register. The energy cost of a MAC varies with hardware, but an order-of-magnitude estimate for a digital implementation is 1 pJ (26, 27). Given that $k_{\text{B}}T$ at 300 K is roughly $4\times 10^{-21}J=4\times 10^{-9}\mathrm{pJ}$, each MAC costs about $2\times 10^{8}\;k_{\text{B}}T$. The teacher neural network has a 784–32–32–10 architecture, which involves 26,432 MACs. The energy cost of evaluating the neural network, for one MNIST digit, is therefore about $5\times 10^{12}\;k_{\text{B}}T$.

If realized in hardware, the energy cost of the thermodynamic computer would be many orders of magnitude smaller. We can estimate this cost by considering the energy scales of different elements of the computer.

First, the inputs. The input to the thermodynamic computer consists of 784 pixels, each represented by a number of order unity on the scale of $k_{\text{B}}T$. In total, therefore, the energy scale of the input is of order $10^{3}\;k_{\text{B}}T$. If each pixel value must be physically replicated across its downstream connections—which may be necessary for some hardware designs but not others—then this cost would increase to about $10^{5}$ *k*B*T*.

**Fig. 3.**
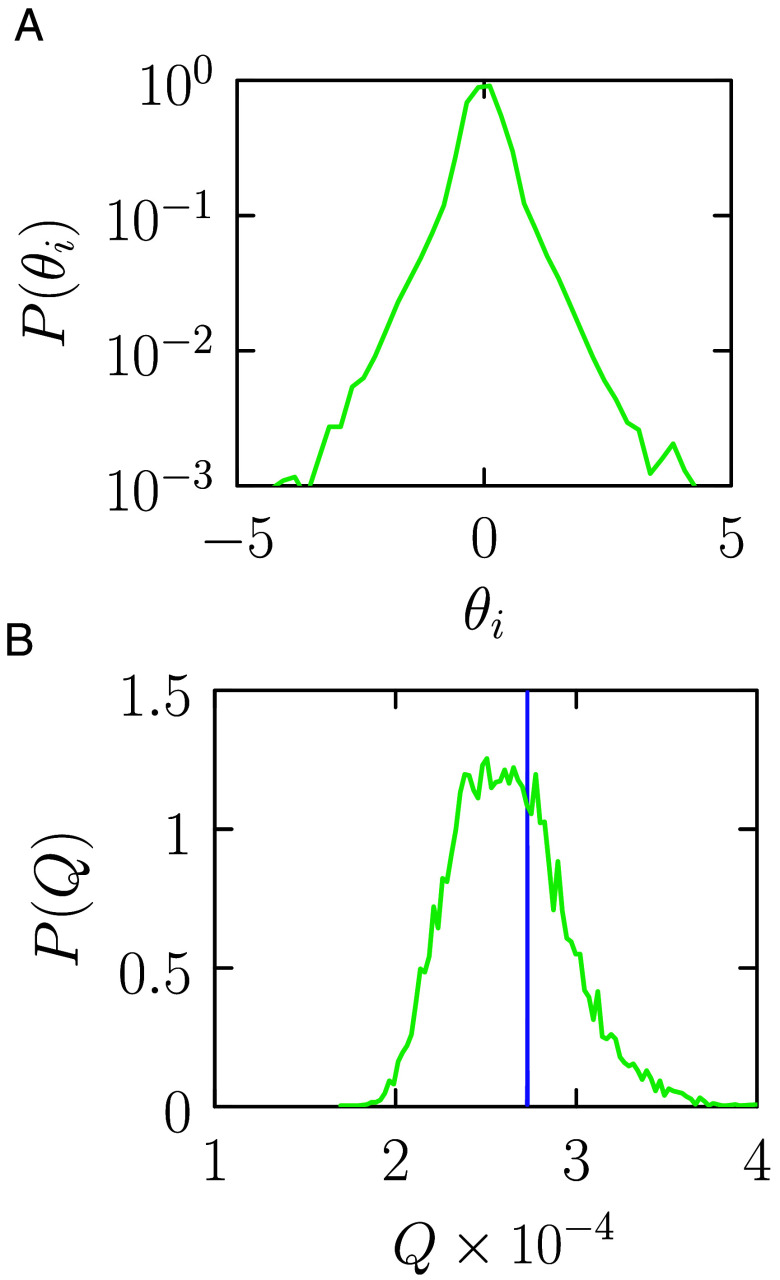
Histograms of (*A*) the couplings of the trained thermodynamic computer and (*B*) the heat it emits while classifying an MNIST digit. In (*B*), the green distribution is taken over the 10,000-member test set, while the blue distribution is taken over 10,000 trajectories using a single test-set digit. Both horizontal axes are given in units of $k_{\text{B}}T$. The overall energy scale of a hardware version of the thermodynamic computer performing MNIST inference would be about $10^{5}k_{\text{B}}T$. By contrast, a *single* multiply-accumulate operation in a neural network using digital hardware costs about $2\times 10^{8}\;k_{\text{B}}T$, and MNIST inference using the teacher neural network costs about $5\times 10^{12}\;k_{\text{B}}T$.

Second, the couplings. In Fig. 3*A* we show a histogram $P(\theta_{i})$ of the values of the 63,143 parameters $\theta_{i}$ of the trained thermodynamic computer. The horizontal axis is given in units of $k_{\text{B}}T$. The distribution is far from Gaussian, with a sharp peak around zero and broad shoulders, reflecting the heterogeneous interactions required to reproduce the neural network’s activations. The typical scale of the couplings is about $k_{\text{B}}T$. In the worst-case scenario, if all couplings have to be supplied via external work during inference, then the associated energy budget would be of order $10^{5}\;k_{\text{B}}T$. However, in existing hardware realizations of thermodynamic computers the couplings are fixed physical parameters, and no external work is required to supply them during inference (5, 6). In this case the energy budget for the couplings is zero.

Third, we can measure the heat *Q* dissipated to the thermal bath during inference, i.e. during a trajectory of the trained computer when presented with an MNIST digit. The heat emitted during a trajectory ${\left\{x\left(t_{k}\right)\right\}}_{k=0}^{K}$ is

[13] $$\begin{array}{c} Q=\sum_{k=0}^{K-1}[V_{\theta }\left(x\left(t_{k}\right)\right)-V_{\theta }\left(x\left(t_{k+1}\right)\right)], \end{array}$$

where $V_{\theta }\left(x\right)$ is given by Eq. **2**. In Fig. 3*B* we show a histogram P(Q) of the heat emitted while classifying an MNIST digit, taken over all 10,000 digits of the MNIST test set (green). The horizontal axis is given in units of $k_{\text{B}}T$. The heat emitted during inference is of order $10^{4}\;k_{\text{B}}T$. We also show (blue) the histogram of heat values for 10,000 trajectories using a single MNIST digit. This distribution is much narrower, and on the scale of the figure appears as a single peak. The comparison demonstrates that the width of the green distribution is set primarily by the differing energetic costs of inference for different input digits, rather than by stochastic fluctuations between trajectories of the same digit.

These three considerations suggest that a hardware implementation of our thermodynamic computer could perform inference with an energy cost of order $10^{5}k_{\text{B}}T$, giving a thermodynamic advantage [**12**] of more than $10^{7}$.

Note that the cost of training the thermodynamic computer digitally is considerably greater than that of training the digital neural network. One training trajectory in our thermodynamic computer simulation requires $K=t_{f}/\Delta t=200$ Langevin steps, and each step requires evaluating interaction forces and accumulating gradients over all $6.3\times 10^{4}$ model parameters, corresponding to ≈4 ×$10^{7}$ MACs per training digit. By comparison, one forward–backward pass of the 784–32–32–10 neural-network teacher requires about $5.3\times 10^{4}$ MACs. Both the thermodynamic computer and the neural network were trained using about $10^{6}$ passes through the training set, implying an additional training cost for the thermodynamic computer of about $4\times 10^{13}$ MACs ($7\times 10^{21}\;k_{B}T$). With an energy saving of ≈5 ×$10^{12}\;k_{B}T$ per inference, approximately $10^{9}$ inferences would be required to amortize this additional digital training cost, after which the thermodynamic device provides a net energetic advantage. As in conventional machine learning, training is typically performed once, while inference may be performed many times, and so the energetic benefit grows with continued use of the trained device.

Finally, the energetic comparison presented here considers conventional digital computation as a reference point. Other paradigms, such as quantum computing, pursue energy efficiency through different physical mechanisms. In particular, present-day quantum computers rely on coherent quantum evolution implemented in hardware operating at millikelvin temperatures, requiring dilution refrigeration and extensive classical control electronics. The energetic cost of maintaining these cryogenic environments and control systems dominates the system-level power consumption. The potential energetic advantage of thermodynamic computing comes from allowing room-temperature dissipative physical relaxation to perform computation.

## 4. Conclusions

We have shown that gradient descent can be used to train a thermodynamic computer to perform computations on a clock, at fixed observation times and in general out of equilibrium. In a recent paper we trained a model thermodynamic computer using gradient descent to implement a generative model (13). Here, we have extended this approach, showing that gradient descent can also train a thermodynamic computer to mimic the behavior of a target neural network trained to perform an arbitrary calculation. The training scheme follows a teacher-student framework: A neural network provides reference activations, which are approximated by idealized Langevin trajectories. By differentiating the Onsager-Machlup functional, we maximize the likelihood that the thermodynamic computer generates these idealized trajectories, thereby shaping its dynamics to reproduce the target computation. We demonstrated this method on an image-classification task.

We had previously shown that a genetic algorithm can be used to train a thermodynamic computer to perform machine-learning tasks (12). Genetic algorithms are as effective as gradient descent, able to reach similar values of loss, but are slower and more costly computationally: For the 10-class MNIST problem, the genetic algorithm required the generation of about $10^{13}$ trajectories; for GD, the number is fewer than $10^{6}$. One advantage of GAs, however, is that they can be applied directly to a device in hardware (28). One could therefore combine these methods, training a digital model of a thermodynamic computer using gradient descent, and, if necessary, retraining its hardware realization using a GA. The latter step is potentially useful if the device production process results in a stochastic distribution of component characteristics, sometimes called device noise.

We also estimated the energy costs associated with the teacher neural network and a hardware implementation of the trained thermodynamic computer, and concluded that the thermodynamic advantage—the ratio of the digital and thermodynamic energy costs—exceeds seven orders of magnitude. This simple example illustrates the considerable potential of thermodynamic computing. The neural network is slightly more accurate than the thermodynamic computer, but this gap in performance can almost certainly be narrowed by fine-tuning the computer’s training procedure. For example, it may be possible to construct teacher trajectories that are easier for a student computer to mimic, and the gradient-descent method could be augmented with techniques commonly used in neural-network training, including momentum and weight decay. In addition, there may be neural networks that are more natural teachers of a thermodynamic computer than is a feedforward neural network. For example, there are clear parallels between the temporal evolution of a thermodynamic computer and the iterative updates of a recurrent neural network (RNN). Both systems maintain internal states that are updated as information is processed. Using RNN-style teacher architectures might provide a natural path toward sequential and memory-based computation in future thermodynamic designs.

In the operating regime studied here, the performance associated with single-trajectory inference is comparable to that associated with averages over multiple independent trajectories, indicating that most of the performance gap between the thermodynamic computer and neural network is systematic rather than statistical. This performance gap can likely be narrowed by fine-tuning the computer’s training procedure. For example, it may be possible to construct teacher trajectories that are easier for a student computer to mimic, and the gradient-descent method could be augmented with techniques commonly used in neural-network training, including momentum and weight decay. In addition, there may be neural networks that are more natural teachers of a thermodynamic computer than is a feedforward neural network. For example, there are clear parallels between the temporal evolution of a thermodynamic computer and the iterative updates of a recurrent neural network (RNN). Both systems maintain internal states that are updated as information is processed, and RNN-style teacher architectures may therefore provide a natural path toward sequential and memory-based computation in future thermodynamic designs.

This paper demonstrates the feasibility of applying machine-learning methodology to thermodynamic devices. It establishes gradient descent as a practical training method for thermodynamic computing, opening the door to training energy-efficient thermodynamic hardware to perform the broad range of tasks addressed by modern machine learning.

## 5. Materials and Methods

Materials and methods are the analytic calculations and computer simulations described in the text.

## Data Availability

The code used in this paper can be found at https://github.com/swhitelam/thermo_gd (29).

## Footnotes

^*^Used in this way, a thermodynamic computer resembles a continuous-spin analog of a Boltzmann machine, a statistical mechanical model that represents probability distributions over binary variables (7, 8).

^†^Note that the choice $J_{2}<0,J_{4}>0$ creates bistable units, analogous to the p-spins in the field of probabilistic computing (20). The choice $J_{2},J_{4}>0$ creates units with one stable state, analogous to the s-units of thermodynamic computing (5).
